# Influence of Temperature on Charge Accumulation in Low-Density Polyethylene Based on Depolarization Current and Space Charge Decay

**DOI:** 10.3390/polym11040587

**Published:** 2019-04-01

**Authors:** Guochang Li, Jiaxing Wang, Wang Han, Yanhui Wei, Shengtao Li

**Affiliations:** 1 State Key Laboratory of Electrical Insulation and Power Equipment, Xi’an Jiaotong University, Xi’an 710049, China; Lgc@qust.edu.cn (G.L.); sli@mail.xjtu.edu.cn (S.L.); 2 Institute of Advanced Electrical Materials, Qingdao University of Science and Technology, Qingdao 266042, China; aq203014303@163.com (J.W.); hanwang5438@163.com (W.H.)

**Keywords:** charge accumulation, low density polyethylene (LDPE), thermally stimulated depolarization current, space charge decay

## Abstract

Temperature is one of the key factors affecting space charge accumulation in high voltage direct current (HVDC) cable insulation material. The influence of temperature on charge accumulation in low density polyethylene (LDPE) has been investigated with a combined thermally stimulated depolarization current (TSDC) method and pulsed electro-acoustic (PEA) method. The experimental results indicate that there exists a transition temperature region of charge accumulation around 50 °C. The total accumulated charges all firstly increase and then decrease with the increasing polarization temperature under three typical polarization electric fields, and they have more accumulated charges in LDPE around 50 °C. The phenomenon has a close link with the dynamic processes of charge trapping and de-trapping, which were verified by TSDC results. At room temperature, the trapped charges are difficult to release from the traps, and these homocharges near the cathode can depress the further injection of the charges. More charges can be injected from the electrodes with the increase of temperature, while the charge migration is relatively lower before 50 °C, leading to more accumulated charges. When the temperature exceeds around 50 °C, the molecular movement is accelerated which can enhance the hopping probability of charges between the adjacent traps, resulting in few accumulated charges.

## 1. Introduction

Space charge accumulation in insulation material is one of the key factors that threatens the safe operation of high voltage direct current (HVDC) cable [1,2,3,4,5]. Charge accumulation in insulation material can cause local electric field distortion, leading to material degradation and aging, or worse, material breakdown may occur [6,7,8,9]. Temperature is an important factor that affects charge movement behaviors in the material; hence, it is therefore important to deeply understand the influence of temperature on charge accumulation characteristics in polyethylene.

Charge accumulation in polymer mainly depends on two physical processes: charge generation and charge dissipation. For the first process, space charges are mainly derived from electrode injection and impurity ionization [10]. The injected charges can be estimated via the Schottky thermal emission model and field emission model. For the second process, charge dissipation behavior in the material can be expressed by the charge trapping and de-trapping model [11,12] and the charge hopping transport model [13]. Temperature and electric field are key parameters affecting the above physical processes in the actual application. At present, the coupling effect of temperature and electric field on the charge accumulation in polyethylene are not detailed in the actual application. There have been many studies regarding the charge transport model and numerical calculation [14,15,16,17,18]. The numerical calculation methods have some limitations, owing to the uncertainty of micro-parameters. The charge characteristics in the material for the actual application cannot be accurately reflected only by the calculation method.

The pulsed electro-acoustic (PEA) method has been widely adopted to observe space charge behaviors intuitively in polymer films in last two decades. In recent years, some works have been done on the effect of temperature gradient on space charge distributions in polymer [16,17,18], which are beneficial to understand the charge behaviors in the material. However, the disadvantage of PEA devices is that the data repeatability is subpar, owing to the influence of the specimen condition and the measuring environment. Thermally stimulated theory was initially proposed to study the released charges in electrets, which has been developed to a mature method to study the trapped charges in polymer [19]. Thermally stimulated discharge current can be measured accurately using the integrated measuring system. At present, the most mainstream thermally stimulated measurement device is the Novocontrol system. However, the maximum polarization voltage of the system is only 1 kV, so it cannot be used to study the polarization process under the strong electric field. To fully reflect the charge accumulation in the material, different characterization methods should be considered.

In this work, a polarization and depolarization system has been designed to study the effects of temperature on charge accumulation in low density polyethylene (LDPE) with a combined thermally stimulated depolarization current (TSDC) method and PEA method. Firstly, the polarization process was carried out in a temperature control chamber, the advantage being that it can satisfy the polarization process under a broad temperature range and a wide electric field range. After that, the depolarization process was characterized respectively by thermally stimulated depolarization current and space charge decay. Thus, the effects of temperature on charge accumulation in LDPE can be fully reflected. Compared with the traditional TSDC and PEA methods, the difference here is that the polarization treatments are performed within a temperature controlled device.

## 2. Experimental Method and Setup

**Figure 1:**
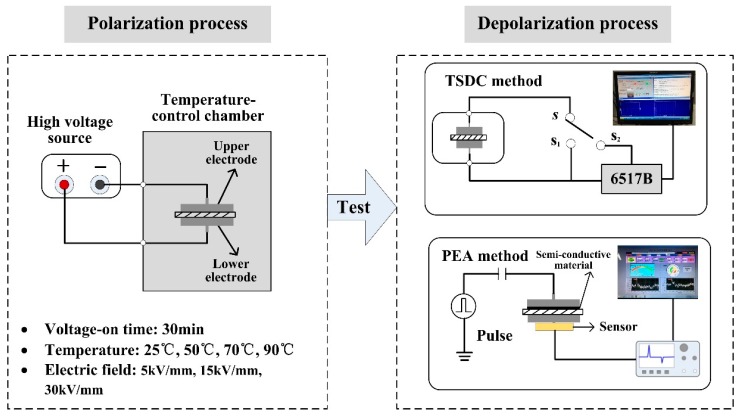
Schematic diagram of experimental setup and conditions: (**a**) Polarization process performed within a temperature controlled device; (**b**) depolarization process implemented by TSDC method and PEA method.

The difference between the experiments conducted here and the regular TSDC and PEA methods is that the polarization treatments were performed within a temperature controlled device. Figure 1 shows a schematic diagram of the experimental method and setup. The polarization system consisted of a high voltage source, a temperature control chamber, and test electrodes. The polarization process was reflected by two systems. The first was a thermally stimulated depolarization current (TSDC) measurement system, which contained an electrometer 6517B, a temperature control chamber, test electrodes, and data acquisition software. The second was a pulsed electro-acoustic (PEA) measurement system, which contained a pulse source, sensor, test electrodes, and data acquisition software.

In the experiments, four typical temperature values (25, 50, 70, and 90 °C) were set. In order to compare and analyze charge injection and transport properties, three electric fields were set: 5, 15, and 30 kV/mm. Pure low-density polyethylene (LDPE) was used in order to study the intrinsic charge characteristics and reduce the influence of external additives. The specimen was fabricated by the melt-compounding method. Firstly, the raw material was implemented by the plate vulcanizing press at 13 °C for 10 min with 10 MPa. Secondly, the specimen was cooled for 10 min with 10 MPa. Finally, LDPE films of 300 μm were obtained, and the specimens were sputtered the metal electrode prior to the test. The polarization process was carried out in a temperature control chamber, as shown in Figure 1a. A pair of plate electrodes with diameters of 45 mm was used to apply voltage to the specimen; the voltage-on time was set to 30 min. After the polarization, the specimen was transferred under open-circuit conditions using insulating tweezers, with the operational process taking 1 min. The depolarization current was measured by the TSDC device. The specimen was placed in a small vacuum chamber, the cylinder structure having a diameter of 100 mm and a height of 250 mm. The heating rate was set at 1 °C/min, and the temperature was set from 20 to 90 °C, with the temperature accurately controlled and measured by the integrated system. Prior to TSDC measurement, the specimen was firstly short-circuited for 1 min to remove the leakage current on the specimen surface, the process being carried out by the integrated system. This was followed by the specimen being removed for space charge decay measuring. The specimen was placed between the upper electrode and the bottom electrode. The charge signal was measured by the bottom sensor of the specimen when the pulse was applied to the specimen. It was noted that no voltage was applied to the sample, and only high voltage pulse power was applied. The pulse width was 5 ns, the pulse voltage 200 V, and the charge decay time 1 h.

## 3. Experimental Results and Analysis

### 3.1. Depolarization Current Properties

**Figure 2:**
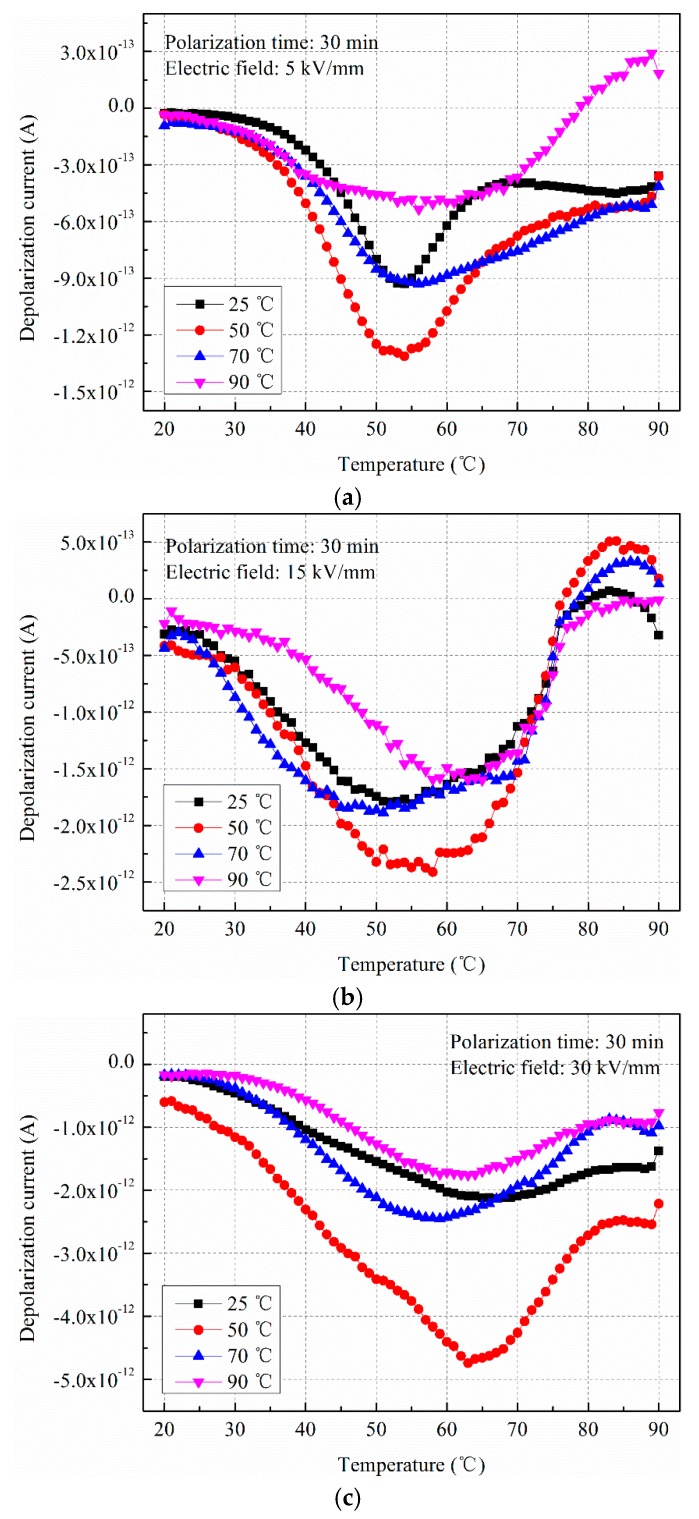
The depolarization current curves in low density polyethylene (LDPE) polarized by different temperatures and electric fields. (**a**) 5 kV/mm; (**b**) 15 kV/mm; (**c**) 30 kV/mm.

Different combinations of temperature (25, 50, 70, and 90 °C) and electric field (5, 15, and 30 kV/mm) were performed. Figure 2 shows the depolarization current curves in LDPE polarized by different temperatures and electric fields.

It can be shown from Figure 2 the depolarization current gradually increases with the increasing depolarization temperature, peaking at a certain temperature because the trapped charges regain enough energy by the thermal function and return to the free charges. Moreover, the positions of the peaks at different polarization temperatures change only slightly around 53 °C, indicating that trap levels are basically the same. For different polarization temperatures, the accumulated space charges in LDPE are different. The accumulated charges firstly increase and then decrease with the increasing polarization temperature, and the maximum current reaches −1.30 × 10^−12^ A at 50 °C under 5 kV/mm.

Figure 2b shows the depolarization current under stress at 15 kV/mm. The maximum depolarization current also appears around the polarization temperature of 50 °C. Moreover, it can be seen that a reverse current occurs when the depolarization temperature exceeds 80 °C; this may be caused by the thermal stimulation. Generally, the reverse current is called an “empty peak”, which does not have actual significance for the analysis of material properties. When the polarization electric field is 30 kV/mm, the trend of the depolarization peak current with polarization temperature follows the same variation trend. The current rises dramatically, and the value reaches −4.74 × 10^−12^ A at 50 °C, as shown in Figure 2c.

Comparing above three cases, it can be observed that the changes in depolarization current present a consistent trend. That is, the accumulated charges in LDPE first increase and then decease with the increasing polarization temperature, and the maximum accumulated charges all appear around a polarization temperature of 50 °C. The phenomenon is closely related to the charge generation process and the charge dissipation process during applied voltage. For different polarization temperatures, the charge generation rate and dissipation rate are different, which will be discussed later.

### 3.2. Space Charge Decay Properties

**Figure 3:**
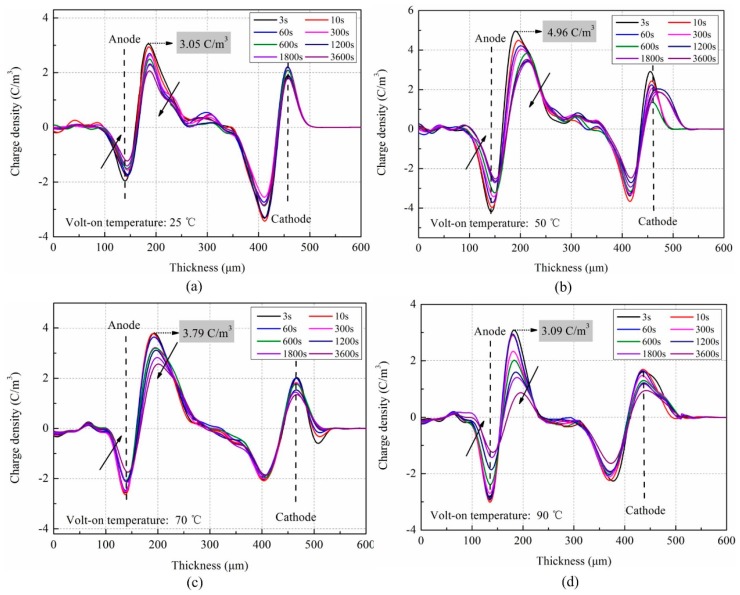
Space charge decay profiles in LDPE polarized at different temperatures. (**a**) 25 °C; (**b**) 50 °C; (**c**) 70 °C; (**d**) 90 °C.

To compare and verify the experimental phenomenon obtained by the TSDC method, space charge decay of LDPE was measured by the PEA method. In the measurement, the polarization conditions remained the same as with the TSDC method. Figure 3 shows space charge decay profiles of LDPE at 25 °C under 30 kV/mm at four typical polarization temperatures.

It can be seen from Figure 3 that the charges near the two electrodes rapidly decay after the removal of voltage, inducing the reverse peak charge. Overall, the space charge decay trends follow the same rules at different polarization temperatures. The position and negative peak charges inside the material occur at a distance of about 50 μm from the specimen surface. The charge migration distance inside the material can be estimated by the charge mobility. Assuming the charge mobility of LDPE is 10^−15^ m^2^/(V·s), the charge migration distance after 1800 s can be calculated at about 54 μm under an applied electric field of 30 kV/mm. This is consistent with the experimental results. Moreover, part of charges captured by the deep traps inside the material is retained after 3600 s. It is difficult to release against the trap sites at 25 °C.

Comparing space charge decay amounts at different polarization temperatures, it can be observed that the accumulated charges are always greatest at 50 °C, both initially and at the end of the experiment. The phenomenon is consistent with the conclusion obtained by the TSDC method. It can be illustrated by the peak charge density near the anode; the charge densities are respectively 3.05, 4.96, 3.79, and 3.09 C/m^3^ at the initial time. It is clear that the accumulated charge around 50 °C is higher than the other three polarization temperatures.

## 4. Discussion

**Figure 4:**
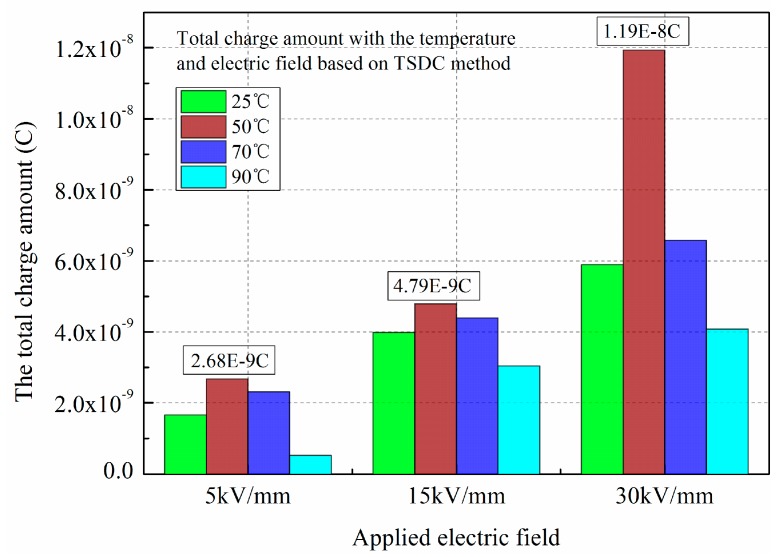
The total charge amount versus the polarization temperature and electric field. Obtained by TSDC method.

**Figure 5:**
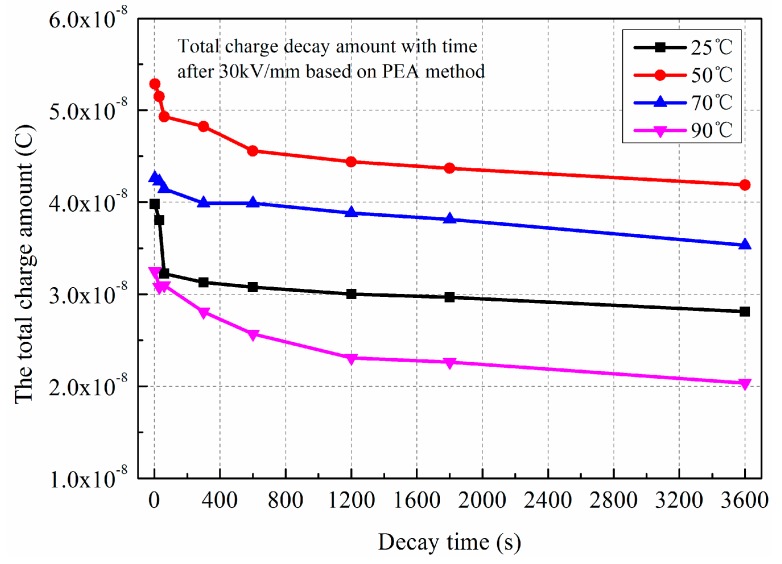
The total charge decay amount versus the polarization temperature under 30 kV/mm. Obtained by PEA method.

To further analyze effect of polarization temperature on charge accumulation in LDPE, the amounts of total charges have been calculated based on the experimental results of TSDC and PEA. Figure 4 shows the total accumulated charges versus the polarization temperature and electric field obtained by the TSDC method. Figure 5 shows the total accumulated charges versus the polarization temperature under 30 kV/mm obtained by the PEA method.

The total charge amount obtained by the TSDC method can be calculated by the following equation:

(1) $$Q_{1}=\frac{1}{\beta }\int_{T_{0}}^{T_{1}}I(T)\cdot dT$$

where *I*(*T*) is the depolarization current measured by TSDC (A), *β* is the heating rate (°C/min), and *T* is the depolarization temperature (°C).

The total charge amount obtained by the PEA method can be calculated by the following equation:

(2) $$Q_{2}=\int_{d_{0}}^{d_{1}}s\cdot \rho (x)\cdot dx$$

where *s* is the specimen area (μm^2^), *ρ*(*x*) is charge density measured by PEA (C/m^3^), and *x* is the specimen position (μm).

It can be seen that the total charge amounts in LDPE under three electric fields all present the same trends, with the maximum charge occurring around 50 °C. When the electric field is 30 kV/mm, the value reaches 1.19 × 10^−8^ C. By analyzing the space charge decay profiles, the total charge decay amounts can be calculated by the integral operation. The same trends can be obtained, there being more space charge accumulation at 50 °C than at other temperatures. The value reaches 4.19 × 10^−8^ C at the final time of 3600 s. Comparing the experimental results of the two methods, the values of the total charges are approximate.

Comparing Figure 4 and Figure 5, the total charge amount obtained by the depolarization current is slightly lower than that of space charge decay. For the PEA method, the interface charges near the electrodes include the induced charges and injected charges, while only injected charges exist for the TSDC method. Furthermore, space charge decay profiles contain the charges captured by the deep traps; these charges can also be calculated by the integral operation. While the depolarization current reflects the released charges at a certain temperature, for the charges captured by the deep traps, the current cannot be measured.

**Figure 6:**
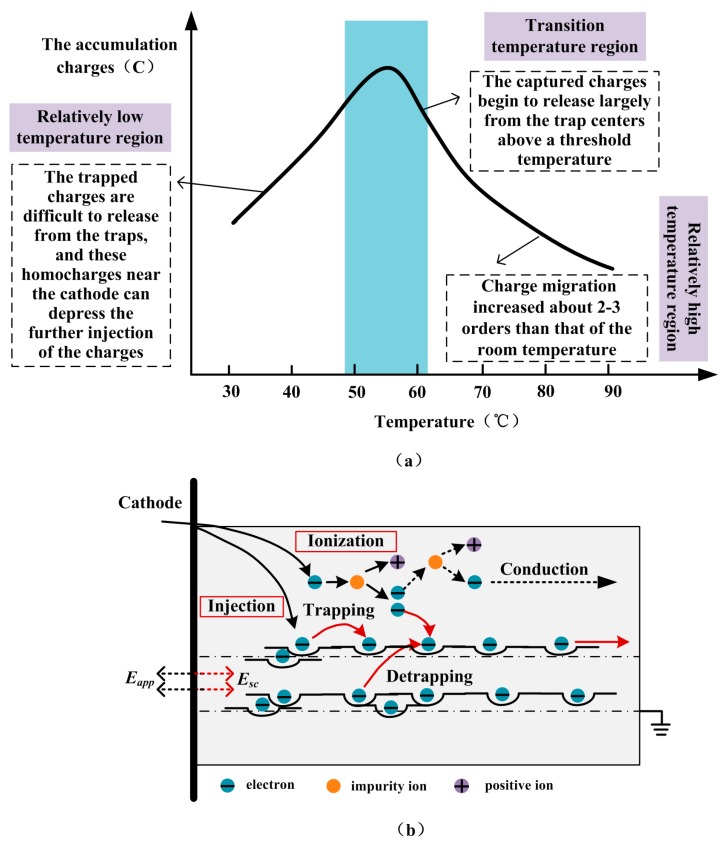
Schematic diagram of the charge accumulation dependence on the polarization temperature. (**a**) The change in trend of the accumulation charges in polymer with the temperature; (**b**) the dynamic processes of charge generation and dissipation in polymer.

The experimental phenomenon obtained by the two methods both indicate that there exists a transition temperature region of charge accumulation in LDPE from 25 to 90 °C. Figure 6 shows the schematic diagram of the charge accumulation dependence on the temperature.

The total charge amount in the material mainly depends on the charge generation rate and the charge conduction rate. For the different compound modes of temperature and electric field, the contribution of the charge generation and conduction on the charge accumulation are different. Trap distribution in the material plays an important role in the charge conduction process. The TSDC method is one of the representative methods to analyze the trap parameters; the trapped charges gradually release from the trap centers with the increase of the temperature, and the current can be measured by the electrometer. The trap level can be calculated by fitting the TSDC curves adopting the initial rise method, which assumes that the TSDC curve is a single frequency relaxation process. Analyzing the TSDC curves in Figure 2a, it can be seen that the maximum depolarization current occurs at around 53 °C, which illustrates that more trapped space charges can regain energy at the temperature range of 50 to 60 °C. The trap levels corresponding to the maximum depolarization current can be extracted, measuring around 0.85, 0.95, 0.90, and 0.80 eV for the four different polarization temperatures in Figure 2a. It can be seen that the trap level in LDPE belongs to the category of a deep trap formed by defects located in the boundary region between the crystalline and the amorphous regions. The value is consistent with the literature of about 1.0 eV at a peak temperature of 47 °C proposed by Ieda [20].

For the relatively low temperature region, the injected charges will be captured by the traps in the material, and most trapped charges cannot obtain enough energy to become the free charges. Overall, the charge migration rate in LDPE is very low, about 10^−20^ S/m [13]. The accumulated charges are not so many at 25 °C, because the injected charges will be captured by the traps near the electrodes. These homocharges will form a local electric field *E_sc_* which is opposite to the applied electric field *E_a_*. These will reduce the interface electric field and weaken further injection of charges, resulting in a relatively small amount of accumulated charges. With the increase of temperature, the interface injection barrier will be reduced, and more charges can be injected from the electrodes, resulting in an increased charge accumulation amount. There are more accumulated charges around 50 °C. Beyond the transition temperature, the molecular movement is accelerated, which can enhance the hopping probability of charges between the adjacent traps [20]. The captured charges begin to be released largely from the traps. The charge migration rate will dramatically rise by two or three orders of magnitude at 90 °C, reducing the charge accumulation in LDPE. The inflection temperature observed in LDPE is in good agreement with the observations of Boudou [14]. The annealing treatment can cause a change in the polyethylene morphology and further affects the charge conduction. At temperatures of 45 °C and above, an exothermic peak was observed by differential scanning calorimeter (DSC) measurements, which shows a morphological modification of the material and that the charge detrapping process is facilitated.

## 5. Conclusions

Thermally stimulated depolarization current and space charge decay have been measured to characterize the charge accumulation in LDPE. Consistent rules have been obtained by the two methods. There exists a transition temperature region of charge accumulation in LDPE around 50 °C. It can be found that the accumulated charges all initially increase and then decrease with increasing temperatures under three typical electric fields, and the inflection temperature appears to be around 50 °C. For relatively low temperatures, the trapped charges are difficult to release from the traps, and these homocharges near the cathode can depress the charges injection. Therefore, the accumulated charges amount in the material is relatively small at 25 °C. More charges can be injected from the electrodes with the increase of temperature, while the charge migration is relatively lower before 50 °C, leading to more accumulated charges in the material. When the temperature exceeds about 50 °C, the charge conduction will be rise dramatically, owing to the morphological changes of polyethylene, and little accumulated charges are left.
